# Association between smoking status, toxicity and survival in the checkpoint inhibitor immunotherapy

**DOI:** 10.3389/fonc.2024.1459608

**Published:** 2024-11-25

**Authors:** Anna Rudzińska, Pola Juchaniuk, Jakub Oberda, Kamila Krukowska, Sylwia Krzyśkowska, Eliza Kuchta, Anna Rodzajewska, Mariola Janiszewska, Katarzyna Szklener, Katarzyna Machulska-Ciuraj, Monika Rychlik-Grabowska, Aleksandra Urniaż, Rafał Urniaż, Sławomir Mańdziuk

**Affiliations:** ^1^ Department of Clinical Oncology and Chemotherapy, Medical University of Lublin, Lublin, Poland; ^2^ Department of Medical Informatics and Statistics with e-Health Lab, Medical University of Lublin, Lublin, Poland; ^3^ OncoCDx Research Center, London, United Kingdom

**Keywords:** cancer immunotherapy, immunotherapy toxicity, immune checkpoint inhibitors, immunotherapy adverse effects, smoking

## Abstract

**Introduction:**

Immune checkpoint inhibitors (ICIs) have revolutionized cancer therapy by enhancing T-cell-mediated immune responses against tumors. However, their use can lead to immune-related adverse events (irAEs), impacting patient outcomes.

**Methods:**

This single-center, observational study investigates the relationship between immune-related adverse events (irAEs) and survival outcomes in 151 patients treated with ICIs, with or without chemotherapy, at the Department of Clinical Oncology and Chemotherapy in the Independent Public Hospital No. 4 in Lublin. Statistical analyses were performed using the log-rank test, and multivariable Cox proportional hazard model (p < 0.05).

**Results:**

IrAEs were observed in 38% of patients, with the most common being thyroid dysfunction (11.9%) and dermal toxicity (6.6%). Individual toxicity groups presented similar median values of “pack-years”, suggesting that smoking did not have a direct impact on the degree of toxicity. No relationship between the number of “pack-years” and the time of occurrence of toxicity symptoms and the number of toxicity sites was found. Smoking status did not have a moderating effect on the toxicity parameter in survival analysis (OS) and progression free survival analysis (PFS). Pack-years of smoking significantly impacted both OS (HR = 1.01, p = 0.014) and PFS (HR = 1.01, p = 0.011).

**Disscusion:**

The results suggested that smoking, measured in pack-years, had no appreciable effect on the amount of toxicity experienced by patients and no correlation between smoking status, irAEs and efficiency of the treatment was found. Despite results not reaching statistical significance, other potential mechanisms by which smoking may influence cancer treatment cannot be ruled out.

## 1 Introduction

Tobacco intake has been proved as an important and common risk factor for multiple cancer development (1, 2). The risk of cancer for smokers is drastically heightened than for never smokers (1). Intensity of smoking, i. e. the number of cigarettes smoked per day and the number of years of smoking is also an important risk factor of cancer and the more aggressive course of illness (1, 3).

List of malignancies including head and neck, pharynx, larynx and tracheal, esophageal, gastric, colorectal, hepatocellular, pancreatic, cervical, kidney, bladder or urothelial carcinoma and acute myeloid leukemia are strongly linked with positive smoking status and described as smoking-related (1). Lung cancer however has the strongest connection to tobacco usage and is found to be the cancer most commonly developed due to excessive smoking with a development risk over 20 times higher in smokers than non-smokers (3). The smoking intensity has been proved to significantly influence the cancer risk, progression risk and the death risk (1, 3). Smoking cessation after the cancer diagnosis has also been found to positively influence patients survival (4).

Smoking has a documented influence on the molecular cell alterations, oftentimes leading to more aggressive and treatment resistant tumors in comparison with non-smokers (2, 4). Smokers population is characterized by the heightened tumor mutation burden (TMB), a factor considered a predictive factor of the immunotherapy response (2, 5, 6). Alterations offer possible target sites to the novel therapeutics, however they are also oftentimes linked with worsened performance status and lowered survival outcomes (7). *TP53* and *KRAS* represent typical mutations for the heightened tobacco consumption and are considered as negative prognostic factors (8, 9). This warrants the role of smoking exposure to the course of the illness and clinical response to the various forms of anticancer therapy, including Epidermal Growth Factor Receptors-Tyrosine Kinase Inhibitors (EGFR-TKIs), immunotherapy and platinum-based chemotherapy (4, 10–12).

In our work we decided to investigate the possible relations between smoking status of the patients treated with immune checkpoint immunotherapy with the occurrence rate of the immunologic adverse effects and treatment efficacy.

## 2 Materials and methods

This single-center, observational study was conducted using medical records of the 151 patients with any solid tumor treated with at least one dose of immunotherapy with or without chemotherapy in the Department of Clinical Oncology and Chemotherapy in the Independent Public Hospital No. 4 in Lublin between 2019 and 2023. Data cut-off date was 26 April 2023. All examined patients were adults (>18 years old), with varying cancer subtypes- predominantly NSCLC. PD-L1 expression results in 97 patients varied between 0% and 100%, in the remaining patients PD-L1 expression scores were unattainable. Immunotherapy administered included pembrolizumab, nivolumab, atezolizumab, avelumab, durvalumab and ipilimumab in addition to PD-1 inhibitor. Chemotherapy regimens consisted of platinum in combination with other drugs, such as pemetrexed, docetaxel, gemcitabine, or paclitaxel/nab-paclitaxel. We collected patient baseline clinical data through electronic medical records, including age, sex, cancer stage, histology, differentiation, smoking history including smoking status and pack years, TNM classification, line of therapy, treatment type, clinical response, time of onset of the irAEs, type of the irAEs (organ-specific), grade of the irAEs, overall survival (OS) and progression-free survival (PFS).

Patients’ irAEs were defined based on pathological proof, laboratory results and clinician decision after excluding other causes. Toxicities were graded by physicians based on Common Terminology for Adverse Events criteria, v4.0 (CTCAE v4.0). In patients receiving immunotherapy with chemotherapy or who received chemotherapy as a previous treatment line, we distinguished between immunotherapy-related and chemotherapy-related adverse events based on the differences in the toxicity spectrum (incidence rate and treatment specific adverse effects) and the time of toxicity onset. Hematological disorders and neuropathy were the most excluded adverse effects.

Tumor response was evaluated by the tomography scan results using the RECIST (Response Evaluation Criteria in Solid Tumors) 1.1 criteria. PFS was defined from the first administration of the immunotherapy until the disease progression, unacceptable toxicity resulting in the change of treatment line, death, or follow-up cut-off date. OS was defined as from the first day of ICI treatment administration of the immunotherapy until death, or follow-up cut-off date. This study was approved by the Medical University of Lublin institutional review board (No KE-0254/198/10/2022).

The data distribution was tested for normality with the Shapiro-Wilk test. The irregularity of the distribution allowed for the utilization of non-parametric statistical methods for the analyzed variables. To compare the survival time between groups, the log-rank test has been used. In the context of analyzing the impact of multiple factors on the survival time, the multivariable Cox proportional hazard model was used. The significance of the model and individual variables were investigated by tests: likelihood ratio test, Walda and score. Before using the Cox model, the proportional hazard test was evaluated.

The effect size for individual variables was expressed using the hazard ratio (HR). The multivariable Cox proportional hazard model fitting was rated by using the R² Nagelkerke factor, that provides information about the proportion of variances in survival times explained by the models.

For assessing the association between two continuous variables, the Spearman’s rank correlation coefficient was used. The statistical significance of the correlation coefficient was computed using an asymptotic approximation of the distribution *t*.

The significance of differences between two or more groups with non-normal distribution was estimated with the ANOVA Kruskal-Wallis test. In terms of effect size, the measure of epsilon squared was calculated. Every statistical analysis results were presented with an adequate significance level, which enabled the assessment of the credibility of the observed relationships and conclusions drawn from the study.

The analysis was performed using the statistic language R (version 4.3.1; R Core Team, 2023), in the Windows 10 pro 64 bit system (compilation 19045), using the *car* packages (version 3.1.2; Fox J, Weisberg S, 2019), *sjPlot* (version 2.8.15; Lüdecke D, 2023), *parameters* (version 0.21.3; Lüdecke D et al., 2020), performance (version 0.10.8; Lüdecke D et al., 2021), report (version 0.5.7; Makowski D et al., 2023), *ggsurvfit* (version 1.0.0; Sjoberg D et al., 2023), *gtsummary* (version 1.7.2; Sjoberg D et al., 2021), *survival* (version 3.5.5; Therneau T, 2023), *ggplot2* (version 3.4.4; Wickham H, 2016), *readxl* (version 1.4.3; Wickham H, Bryan J, 2023) and *dplyr* (version 1.1.3; Wickham H et al., 2023).

## 3 Results

In the study group among patients with cancer, the median age was 69.0. Women represented 35.1% (n=53) and men 64.9% (n=98) of the cohort. Ex-smokers constituted nearly 41% and active smokers 26.5% of the group. Non-smokers represented almost 1/3 of the cohort (32.5%). Median of pack-years amounted to 20. Median PD-L1 expression count reached 20%. In most patients immunotherapy constituted for the first (n=69, 45,7%) or second (n=77, 51%) line of the systemic treatment. Among 151 patients, most of them (65.6%, *n =* 99) were treated with the anti-PD-1 antibodies, while the other 34.4% *(n =* 52*)* with anti-PD-L1 antibodies.

Among 151 patients, the most common cancer type was the non-small cell lung cancer (NSCLC) which constituted 78.1% of the cases (*n =* 118). Bladder cancer was the second most prevalent cancer type, representing 9.3% of the population (*n =* 14). The analysis of 115 NSCLC patients disclosed that the most common diagnosed subtype was squamous cell carcinoma (48.7%, *n =* 56) and adenocarcinoma (43.5%, *n =* 50).

Survival status demonstrated that 49.0% patients (*n* = 74) died, and 51.0% (n = *77*) were censored during the analysis meaning that they were still alive or their tracking data were lost. Progression status revealed that 53.0% of patients (n = 80) experienced progression of the disease, and 47.0% (n = 71) had no proof of the disease progression.

Most of the patients (63.6%) had no toxicity symptoms, mild symptoms occurred in 12.6% of the patients. Moderate (17.0%) and severe (6.0%) toxicity were found in 26 and 9 patients. Very severe toxicity was found in one patient (0.7%). Most common site specific toxicity was thyroid toxicity (11,8% n=18).

Characteristics of the study cohort are presented in **Table 1**.

**Table 1:** Demographic characteristics of the patients cohort.

Characteristics	N	Distribution^1^
Age (years)	151	69.0 (64.5, 73.0)*^2^*
Sex	151	
Female		53 (35.1%)
Male		98 (64.9%)
Smoking status:	151	
Active smoker		40 (26.5%)
Non-smoker		49 (32.5%)
Previously smoker		62 (41.0%)
Packyears	151	20.0 (0, 40.0)
PD-L1 expression (%)	97	20 (0-100)
Type of cancer:	151	
NSCLC		118 (78.1%)
Urinary bladder cancer		14 (9.3%)
SCLC		9 (6.0%)
Kidney cancer		7 (4.6%)
Other		3 (2.0%)
NSCLC subtype	115	
Squamous cell carcinoma		56 (48.7%)
Adenocarcinoma		50 (43.5%)
NOS		3 (2.6%)
Pleomorphic cell carcinoma		1 (0.9%)
Large cell carcinoma		5 (4.3%)
Cancer stage		
I		14 (9,3%)
II		22 (14,6%)
III		28 (18,5%)
IV		87 (57,6%)
Metastases status		
Yes		67 (44,4%)
No		45 (29,8%)
Unknown		39 (25,8%)
Treatment:	151	
anti-PD1		99 (65.6%)
anti-PD-L1		52 (34.4%)
Immunotherapy therapeutic		
Atezolizumab		38 (25,2%)
Avelumab		10 (6,6%)
Durvalumab		4 (2,6%)
Nivolumab		28 (18,5%)
Pembrolizumab		71 (47%)
Immunotherapy monotherapy		106 (70,2%)
Immunotherapy plus chemotherapy		42 (27,8%)
Immunotherapy PD-1 plus CTLA-4		3 (2%)
Number of the previous systemic treatment lines:		
0		69 (45,7%)
1		77 (51%)
2 and more		5 (3,3%)
Overall survival (OS), weeks	151	35.6 (21.9, 55.1)
Progression free survival (PFS), weeks	151	26.6 (13.4, 44.4)
Survival status:	151	
Dead		74 (49.0%)
Censored		77 (51.0%)
Progression status:	151	
Progression		80 (53.0%)
No progression		71 (47.0%)
Initial response		
Partial response		33 (21,9%)
Disease stabilization		69 (45,7%)
Progression		24 (15,9%)
Death		20 (13,2%)
Not reached		5 (3,3%)
Toxicity sites	151	
Skin toxicity		10 (6.6%)
Thyroid toxicity		18 (11.8%)
Hepatotoxicity		12 (7.9%)
Kidney toxicity		4 (2.6%)
Fatigue		3 (2.0%)
Arthritis		2 (1.3%)
Number of toxicity sites	151	
None		95 (62.9%)
1		43 (28.5%)
2		10 (6.6%)
3		2 (1.3%)
10		1 (0.7%)
Time of the toxicity onset, cycles	151	0,0 (0,0, 3,0)*^2^*
Time of the toxicity onset, weeks	151	0,0 (0,0, 7,5) *^2^*
Toxicity	151	
None		96 (63.6%)
Mild		19 (12.6%)
Moderate		26 (17.2%)
Severe		9 (6.0%)
Extremely severe		1 (0.7%)

**^1^ n* (%); *^2^ Mdn* (*Q1*, *Q3*) Annotation: N*, sample size; *n*, group size; *Mdn*, median; *Q1*, first quartile (25%); *Q3*, third quartile (75%).

Data analysis of “pack-years”—a term used to describe the number of packs of cigarettes smoked by cancer patients—provided information about the potential relationship between smoking and the severity of cancer treatment toxicity.

### 3.1 Analysis of the effect of pack-years on the severity of toxicity

**Figure 1:**
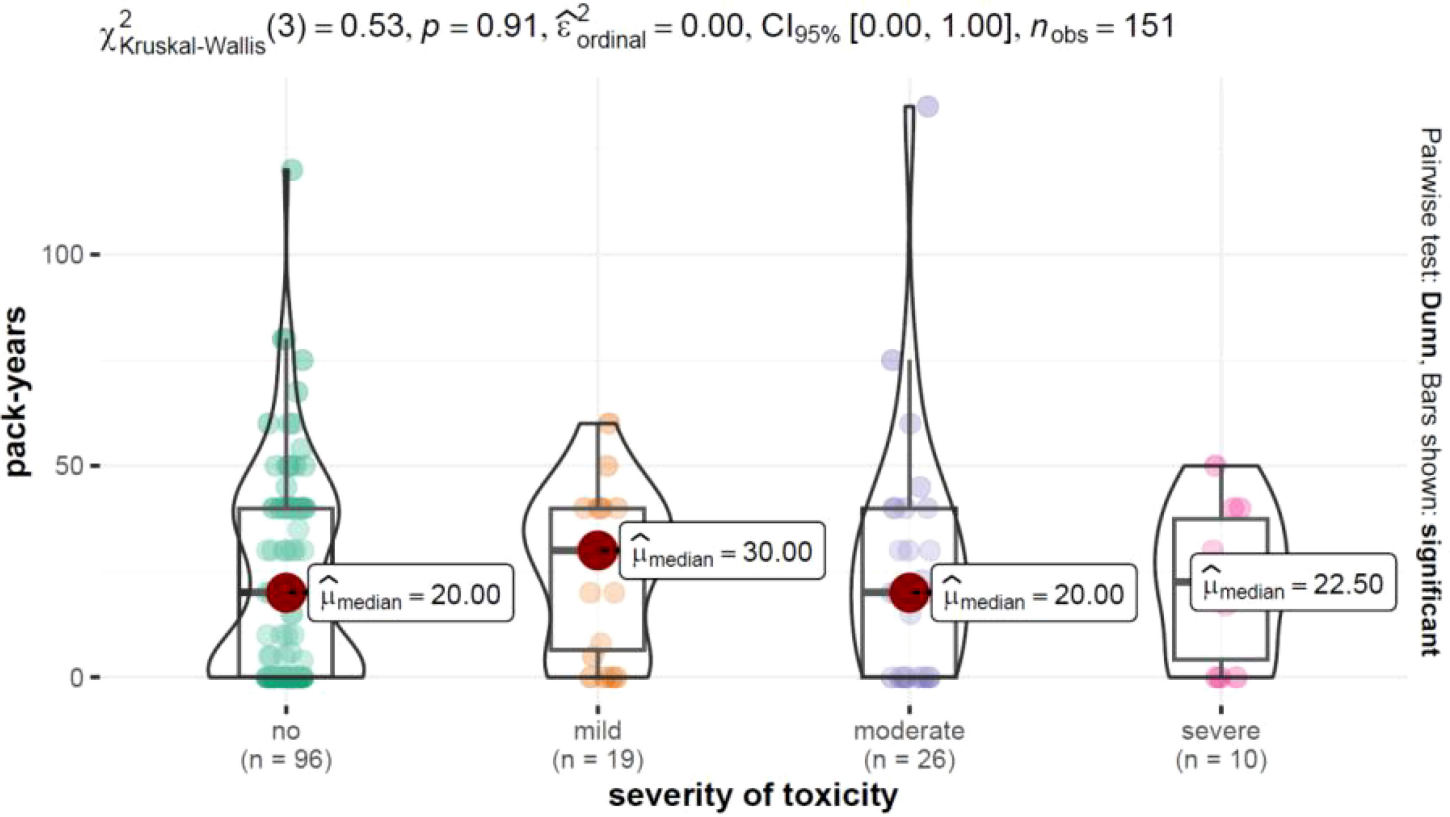
Distribution of pack-years depending on the level of toxicity severity along with results of the test examining intergroup differences.

The study focused on different patients, grouped according to the level of treatment toxicity and divided into four categories: no toxicity (n1 = 97), mild toxicity (n2 = 19), moderate toxicity (n3 = 26), and severe/very severe toxicity (n4 = 10). Visualization of the results shown in **Figure 1**.

The Interquartile range (Q3-Q1) highlighted the variability in the number of pack-years among patients, which indicated individual differences in smoking behavior. The p-value of 0.892 from the Kruskal-Wallis test did not show statistically significant differences in the number of pack-years between groups with different severity of toxicity.

### 3.2 Analysis of the relationship between the number of “pack-years” and the time of occurrence of toxicity symptoms and the number of toxicity sites

Spearman’s rank correlation showing the low rho value of 0.10 and lack of statistical significance between the variables (p = 0.231) suggested that the pack-years variability was not related to the duration of toxicity in a significant way. Spearman’s rank analysis did not show a statistically significant relationship between the number of pack-years and the number of toxicity foci with the weak correlation (rho = 0.05) and lack of statistical significance (p = 545).

### 3.3 Evaluating the moderating effect of the number of pack-years on the toxicity parameter in survival analysis

The analysis used a Cox proportional hazards model to examine the interaction between smoking and toxicity while considering age and sex. The results of the moderating effect are presented in **Table 2**.

**Table 2:** Cox proportional hazards modelof pack-years factor on the connection between toxicity and overall survival (OS), *N_obs_* = 151.

Moderating effect	Overall survival (OS)		
	HR	CI 95%	p
toxicity [mild] × pack-years	0,99	0,96 – 1,02	0,583
toxicity [moderate] × pack-years	1,00	0,98 – 1,02	0,826
toxicity [severe and very severe] × pack-years	0,99	0,95 – 1,03	0,647

*Annotation: Nobs*, number of observations; *HR*, hazard ratio; *CI 95%*, confidence interval 95%; *p*, statistical test *p*-value.

HR values for the interaction between toxicity and pack-years were close to 1.0 and had p-values well above 0.05, indicating that there was no statistically significant evidence that “pack-years” moderated the effect of toxicity on the risk of death.

The analysis used a Cox proportional hazards model to examine the interaction between smoking and toxicity while considering age and sex. The results of the moderating effect are presented in **Table 3**.

**Table 3:** Results of the moderating effect of smoking on the connection between toxicity and overall survival (OS), *N_obs_* = 151.

Moderating effect	Overall survival (OS)		
	HR	CI 95%	p
toxicity [mild] × smoking [in the past]	2,23	0,23 – 21,43	0,488
toxicity [moderate] × smoking [in the past]	0,90	0,19 – 4,33	0,893
toxicity [severe and very severe] × smoking [in the past]	1,40	0,14 – 14,36	0,779
toxicity [mild] × smoking [at present]	5,38	0,47 – 61,65	0,176
toxicity [moderate] × smoking [at present]	2,64	0,47 – 16,12	0,294
toxicity [severe and very severe] × smoking [at present]	4,16	0,37 – 47,16	0,250

*Annotation: Nobs*, number of observations; *HR*, hazard ratio; *CI 95%*, confidence interval 95%; *p*, statistical test *p*-value.

All interactions, for both past and present smoking, had p-values well above the conventional threshold for statistical significance (p < 0.05).

### 3.4 Evaluating the moderating effect of the number of “pack-years” on the toxicity parameter in the analysis of progression-free survival

Similarly to section 2.14, the potential moderating effect of “pack-years” on the relationship between toxicity and PFS in cancer patients was assessed. The results are presented in **Table 4**.

**Table 4:** Results of the moderating effect of smoking on the connection between toxicity and progression-free survival (PFS), *N_obs_* = 151.

Moderating effect	Progression-free survival (PFS)		
	HR	CI 95%	p
toxicity [mild] × pack-years	0,99	0,97 – 1,02	0,691
toxicity [moderate] × pack-years	0,99	0,98 – 1,01	0,558
toxicity [severe and very severe] × pack-years	0,97	0,93 – 1,01	0,190

*Annotation: Nobs*, number of observations; *HR*, hazard ratio; *CI 95%*, confidence interval 95%; *p*, statistical test *p*-value.

HR values for the interaction between toxicity and “pack-years” were close to 1.0 and had p-values well above 0.05, indicating that there was no statistically significant evidence that “pack-years” moderated the effect of toxicity on disease progression.

The results presented in **Table 5** suggested that smoking was not a statistically significant moderator of the impact of toxicity on disease progression (PFS) in the analyzed group of oncology patients with p-values for all tested interactions higher than 0.05.

**Table 5:** Results of the moderating effect of smoking on the connection between toxicity and progression-free survival (PFS), *N_obs_* = 151.

Moderating effect	Progression-free survival (PFS)		
	HR	CI 95%	p
toxicity [mild] × smoking [in the past]	1,16	0,21 – 6,95	0,832
toxicity [moderate] × smoking [in the past]	0,77	0,17 – 3,57	0,736
toxicity [severe and very severe] × smoking [in the past]	0,41	0,04 – 4,30	0,459
toxicity [mild] × smoking [at present]	1,37	0,19 – 9,93	0,758
toxicity [moderate] × smoking [at present]	0,92	0,13 – 6,54	0,934
toxicity [severe and very severe] × smoking [at present]	0,72	0,06 – 9,28	0,804

*Annotation: Nobs*, number of observations; *HR*, hazard ratio; *CI 95%*, confidence interval 95%; *p*, statistical test *p*-value.

### 3.5 Multivariable analysis

The results of the multivariate analysis using the multivariable Cox proportial hazard model to evaluate the effect of different variables on overall survival (OS) and progression free survival (PFS) among patients. Neither ex-smokers, nor active smokers did show any significant difference in death risk compared to non-smokers (*p* = 0.765 and *p* = 0.796). Ex-smokers demonstrated a trend towards an increased risk of the progression (*HR* = 1.90, *p* = 0.071). For active smokers (*HR* = 1.25, *p* = 0.566) no significant impact on the risk of progression was found out. The pack-years were significantly related to the death risk (*HR* = 1.01; *p* = 0.014) and progression risk (*HR* = 1.01, *p* = 0.011).

## 4 Discussion

Cigarette smoking is one of the most established and prominent risk factors for many types of cancers, with a most significant contribution to lung cancer development (1, 3). The impact of tobacco on cellular and molecular changes, including increased tumor mutation burden (TMB), increased PD-L1 expression, microsatellite instability, and the presence of mutations such as TP53 and KRAS, has been widely described in the literature (5, 6, 13, 14). These molecular changes not only enhance tumor aggressiveness but also provide possible targets for new immunotherapies (4). While smoking is generally associated with a poor prognosis, the high PD-L1 expression and TMB commonly observed in smokers would suggest that the smoking history may be relevant to response to immune checkpoint immunotherapy (ICI), particularly for non-small cell lung cancer (NSCLC) (14).

Our study estimated to look at the relationship between smoking history and treatment outcomes, and immunotherapy-related toxicity in cancer patients. Results showed that NSCLC patients with a history of smoking treated with ICIs had better overall survival (OS), progression-free survival (PFS), and response rates compared to never-smokers, possibly because tobacco use is related to higher TMB and PD-L1 levels (14–16). Although a high tobacco exposure level has been recognized as one of the prognostic variables for cancer, our analysis did not show any statistically significant association between smoking history and severity or duration of toxicity in immunotherapy patients. This would suggest that molecular alteration caused by smoking may enhance treatment efficacy without significantly increasing treatment-related toxicity.

Several studies have associated higher TMB and PD-L1 expression—molecular features typically elevated in smokers—with better outcomes in ICI therapy (2, 5, 6). In the present study, smokers showed better OS and PFS outcomes than never-smokers in the NSCLC cohort, which may support the hypothesis that smoking-related alterations could enhance ICI efficacy. These results are consistent with previous reports that high PD-L1 and TMB levels make tumours more recognizable by immune cells and potentially increase the response to ICIs (14, 15). Despite these molecular benefits, smoking history was not associated with increased toxicity, which may indicate that although smoking contributes to responsiveness to immunotherapy, it may not necessarily increase the risk of adverse immunologic reactions in treated patients.

The analysis of pack-years as a potential moderator of toxicity suggested there was no significant association, and that toxicity remained independent of the intensity of smoking. Statistical models, such as Cox proportional hazards analysis, yielded hazard ratios close to 1.0 with p-values greater than the threshold for statistical significance. These findings suggest that the number of pack-years did not have a significant influence on the duration or severity of treatment toxicity due to immunotherapy among our study population. Given the established impacts of smoking on systemic inflammation and pulmonary function, it seemed reasonable to initially hypothesize a direct association between smoking and severity of toxicity. However, the results suggest that other factors, possibly including genetic predispositions, environmental exposures, and comorbidities, may be more important in determining outcomes related to toxicity than the smoking status alone.

Several limitations of the current study have to be addressed while interpreting the findings. The sample size is relatively small: 151 patients, collected from a single institution, which may decrease both the statistical power and the generalizability of the results, increasing the possibility of Type II errors and not capturing the variability that might be present in more heterogeneous collections of patients. Additionally, the single-center design may introduce specific biases due to institutional protocols, regional practices, and patient demographics that may not be typical in other healthcare settings. The observational nature of this study, not involving randomization or a control group, further amplifies the susceptibility to confounding variables, which may influence survival outcomes and adverse events independently of immunotherapy. The sample is also heterogeneous; this includes different subtypes of cancer, mostly NSCLC, and different immunotherapy and chemotherapy regimens. Such heterogeneity makes interpretation difficult, as treatment response and adverse events could vary greatly between different cancer types and by combination of drugs. Lastly, the short follow-up duration of the adverse events may not fully present their spectrum and long-term impact, respectively, limiting the possibility of determining their durability and implications for patient outcomes. Future studies should be carried out with larger, multi-center cohorts and controlled randomized designs, including standardized adverse event assessment with control groups, to enhance the reliability and general applicability of immunotherapy for cancer.

These results are in line with the observation that, although smoking is associated with molecular alterations that may improve immunotherapy efficacy, it does not directly predispose patients to increased toxicity. This has possible clinical implications, in that pack-years may be useful as a prognostic indicator of response to ICI rather than as a predictive biomarker for treatment tolerance. Future studies should therefore consider smoking history as part of risk stratification in immunotherapy protocols, with heavy smokers possibly being subjected to more intensified monitoring for assessment of their response dynamics to ICIs. Such findings await prospective longitudinal studies that will further investigate the complex interactions between smoking, ICI efficacy, and toxicity across a variety of cancer types and treatment settings with genetic and lifestyle factors affecting outcomes.

This study adds to the growing body of literature on the multifaceted role of smoking in cancer immunotherapy and points to the need for focused studies to find out more precisely how smoking affects the efficacy and tolerability of ICIs in cancer treatment.

## 5 Conclusions

The results suggested that smoking, measured in pack-years, had no appreciable effect on the amount of toxicity experienced by patients during cancer treatment in the study group. Although statistical analysis did not demonstrate a direct relationship between smoking and the severity of treatment toxicity, other potential mechanisms by which smoking may influence cancer treatment and the patient’s overall health cannot be ruled out.

## Data Availability

The raw data supporting the conclusions of this article will be made available by the authors, without undue reservation.
